# Comparative effectiveness research

**DOI:** 10.4103/2229-3485.80365

**Published:** 2011

**Authors:** Chitra Lele

**Affiliations:** *Chief Scientific Officer, Sciformix Corporation, Andheri East, Mumbai 400093, India*

The area of Comparative Effectiveness Research (CER) merits significant attention and action in 2011, not only in the US and the Europe Union (EU) where it has been in focus for a couple of years, but also in other parts of the world, including India.

CER focuses on effectiveness of drugs, not efficacy. It addresses the questions of which treatment works best, for whom and under what conditions. Usually, at least two alternatives are compared, including one which is the standard of care. CER focuses on measuring outcomes, risks, and benefits that matter to patients: (a) Efficacy (including symptoms, morbidity, mortality), (b) Safety (reduced interactions leading to fewer and shorter ER visits and hospitalization), (c) lifestyle improvements (Quality of Life, Activities of Daily Living) and (d) cost of treatment.

CER encompasses development, expansion, and synthesis of a variety of data sources to help clinical practitioners make the right evidence-based decision for every patient. Data from RCTs have severe limitations for comparing effectiveness of treatments and in generalization to the real-world setting. Hence, data from electronic health records, retrospective and prospective observational studies, patient registries, pharmacoepidemiological studies, and spontaneous adverse event reporting are also synthesized.

We need to immediately focus on CER in India for the following reasons:

- Disease patterns in India are evolving. There’s little epidemiological data on most disease conditions. Understanding disease profile is a prerequisite to introduction of new treatments.
- Acknowledgment of geographical differences in safety and efficacy of drugs, and regional differences in the standard of care for some conditions necessitates availability of relevant comparative data that are local.
- New drugs are being introduced in India soon after their global launch, with little real-world safety data being available. Hence, postmarketing safety surveillance in India is even more important.
- Industry is looking to replace traditional promotional practices for marketing drugs with research-based evidence.

Collecting and analyzing local observational data from various sources is thus important for introduction and marketing of treatments and for clinical practice. CER is important for all stakeholders––patients, medical practitioners, hospitals, pharmaceutical industry, and medical insurance providers.

I plan to do my bit to raise awareness about CER in India by talking about this in various forums and to various stakeholders. I also plan to help design, conduct, and analyze observational studies through my organization. I also intend to focus on methodological research in the area of risk-benefit analysis and comparing safety of drugs and thus contribute to development of CER.

